# Enhanced brackish water desalination in capacitive deionization with composite Zn-BTC MOF-incorporated electrodes

**DOI:** 10.1038/s41598-024-66023-y

**Published:** 2024-07-01

**Authors:** Amirshahriar Ghorbanian, Soosan Rowshanzamir, Foad Mehri

**Affiliations:** 1 Hydrogen & Fuel Cell Research Laboratory, School of Chemical, Petroleum and Gas Engineering, Iran University of Science and Technology, Narmak, Tehran, 16846-13114 Iran; 2 Center of Excellence for Membrane Science and Technology, Iran University of Science and Technology, Narmak, Tehran, Iran

**Keywords:** Capacitive deionization, Brackish water, Water desalination, Metal–organic framework, Electrode, Chemical engineering, Electrochemistry

## Abstract

In this study, composite electrodes with metal–organic framework (MOF) for brackish water desalination via capacitive deionization (CDI) were developed. The electrodes contained activated carbon (AC), polyvinylidene fluoride (PVDF), and zinc-benzene tricarboxylic acid (Zn-BTC) MOF in varying proportions, improving their electrochemical performance. Among them, the E4 electrode with 6% Zn-BTC MOF exhibited the best performance in terms of CV and EIS analyses, with a specific capacity of 88 F g^−1^ and low ion charge transfer resistance of 4.9 Ω. The E4 electrode showed a 46.7% increase in specific capacitance compared to the E1 electrode, which did not include the MOF. Physicochemical analyses, including XRD, FTIR, FESEM, BET, EDS, elemental mapping, and contact angle measurements, verified the superior properties of the E4 electrode compared to E1, showcasing successful MOF synthesis, desirable pore size, elemental and particle-size distribution of materials, and the superior hydrophilicity enhancement. By evaluating salt removal capacity (SRC) in various setups using an initially 100.0 mg L^−1^ NaCl feed solution, the asymmetric arrangement of E1 and E4 electrodes outperformed symmetric arrangements, achieving a 21.1% increase in SRC to 6.3 mg g^−1^. This study demonstrates the potential of MOF-incorporated electrodes for efficient CDI desalination processes.

## Introduction

The remarkable role of electrodes in enhancing the CDI technology is undeniable, and most attention was paid to improve physical, chemical, and electrochemical properties of electrodes^1–3^. The most important factors enhancing the SRC during CDI are high porosity and specific surface area (SSA), desirable pore size, particle-size distribution, suitable wettability, high electrochemical performance, and physical and chemical stability of electrodes^1,4–6^. For this purpose, the most common and widely used material is AC^7^. However, the utilization of other types of carbonaceous materials, especially environmentally friendly ones (e.g., corn-stalk-based^8,9^ and water hyacinth-based^10^ carbon aerogels) as well as conventional ones (e.g., mesoporous carbon^11^, graphene^12^, and carbon nanotubes^13^), the addition of metal oxide nanoparticles (e.g., TiO_2_^14^, SiO_2_, Al_2_O_3_^15^, and ZnO^16^), and conductive polymers (e.g., polyaniline^17^ and sulfonated polystyrene^18^) to the carbonaceous structures of the electrodes were carried out to achieve a higher specific capacity, lower charge transfer resistance, more hydrophilicity, and better wettability. This leads to improved electrochemical performance in the CDI process^19–21^. In recent years, MOFs have emerged as a type of three-dimensional porous compounds composed of metal ions linked to the organic ligands^22,23^. The incorporation of these materials in the electrodes of some electrochemical systems (e.g., supercapacitors^24^ and electrical absorption processes^25,26^) is a good representation of their unique properties such as porosity, high SSA, hydrophilicity, and desirable physical properties^22,27–30^. However, due to the relatively poor electrical conductivity, high manufacturing cost, and limited direct applications in electrochemical processes reported for most MOFs^26,31,32^, some researchers have fabricated MOF-carbon derived composites to increase the electrical conductivity and porosity of these materials^33^. For instance, Li et al.^34^ fabricated carbon electrodes using Mg-MOF-74 as the precursor material for the CDI process. The resulting electrodes had a SRC of 16.82 mg g^−1^ for a feed solution containing 500 mg L^−1^ of NaCl.

On the other hand, the combination of MOFs and conductive or carbonaceous materials such as AC is known as a suitable solution to not only compensate for the MOFs’ poor conductivity^29^, but also bypass the time-consuming and harsh procedures of Acid-washing and carbonization treatment^23,35^ involved in the fabrication of MOF-derived materials and consequently reduce the manufacturing costs of these electrodes^35^. Wang et al.^36^ prepared ZIF-67 MOF/polypyrrol hybrid electrodes which yielded 11.34 mg g^−1^ SRC from a 584 mg L^−1^ NaCl solution. The organic and metallic parts of these porous materials are combined with each other to makes their use in electrochemical processes very attractive ^36^. In this regard, the type of metal and organic linker involved in the MOF is significant. For example, Benzene-1,3,5-tricarboxylic acid (H_3_BTC) with three functional groups is a frequently used organic linker because of its more coordination mode with metal ions in all three directions and consequently producing higher porosity than another linker^37,38^.

Regarding the effect of metal in MOF-based electrodes in CDI process, a few research studies on metals were employed in MOFs such as Cobalt-MOF-based ZIF-67^36,39,40^, Chromium-based Metal–Organic Framework (Cr-MOF)^41^, Cu-MOF^29^, and Mn-Fe-MOF-based^27^ electrodes. Xu et al.^40^ fabricated hybrid electrodes using ZIF-67 and CNTs which could achieve a SRC of 16.90 mg g^−1^ in a 5 mM NaCl solution. Feng et al.^27^ prepared hybrid electrodes by combining Mn-Fe-MOF with holey graphene in the initial feed of 800 mg L^−1^ NaCl, and the hybrid electrodes led to a SRC of 39.6 mg g^−1^. Zhang et al.^39^ were able to achieve 14.4 mg g^−1^ SRC in a CDI process by the preparation of nanopatterned ZIF-67 MOF electrodes using a feed solution of 5.0 mM NaCl.

Until now, only a few cases have limited the direct use of unmodified or untreated MOFs in CDI electrodes for water desalination. All of these works were performed in last few years, focusing on the use of metals such as Fe, Co, Ni and Cu in the MOF structure in slightly larger quantities of MOFs^27,29,36,39–43^. This poses a cost challenge for scaling up the process^44^. Among the different transition-metal ions involved in MOFs, Zn-BTC MOF represents an attractive choice for MOF-based electrode materials. This is because Zn^2+^ can provide more isostructural porous frameworks^45^, favorable electrical conductivity^46^, low cost, lack of toxicity, high chemical stability in aqueous electrolytes, as well as high energy and charge density^47,48^. Moreover, the easily commercially available H_3_BTC organic ligand facilitates the fabrication of a zinc-based MOF via a facile one-pot synthesis using low-cost starting materials^49^, including zinc nitrate hexahydrate and H_3_BTC under solvothermal conditions. Considering the desirable properties of Zn-BTC MOF, such as excellent water sorption abilities, good stability, and performance over multiple sorption and desorption cycles in aqueous solutions^50–52^, as well as favorable storage and thermal stability up to 200 °C^50–53^, its performance in the CDI process has not been thoroughly investigated. To the best of our knowledge, the utilization of Zn-BTC MOF as well as the evaluation of symmetric and asymmetric electrodes arrangements due to their desirable properties and high charge density is being investigated for the first time in a CDI system. In this study, the aim is to improve the physicochemical and electrochemical properties of conventional carbon electrodes for brackish water desalination by using different proportions of the synthesized Zn-BTC MOF and AC. Therefore, a small percentage of Zn-BTC MOF (ranging from 2 to 10 wt%) was used as an additive to enhance electrode performance. Comprehensive physicochemical and electrochemical characterization tests were performed to analyze the synthesized MOF and fabricated composite electrodes. Furthermore, the desalination performance of the electrodes was investigated in both symmetric and asymmetric arrangements using a CDI cell.

## Materials and methods

### Materials

Zinc nitrate hexahydrate (Zn(NO_3_)_2_·6H_2_O, 99%) and polyvinylidene fluoride (PVDF, MW of 530,000) were obtained from Sigma-Aldrich, Germany. Activated carbon powder (DARCO, BET surface area of 723 m^2^ g^−1^), benzene-1,3,5-tricarboxylic acid (C_9_H_6_O_6_, 95%), N-methyl-2-pyrrolidone (NMP, 99.5%) and ethanol (99.8%) were purchased from Merck Co., Germany. Graphite sheets (500.0 µm thickness) were supplied by Dongbang Carbon Co., China. Carbon cloth was purchased from AvCarb Material Solutions, US. All chemicals were analytical reagent grade and used without further purification.

### Zn-BTC MOF synthesis

Zn-BTC MOF was prepared by a simple solvothermal method^49^ as depicted in Fig. S1. Initially, 1.8 g of Zn(NO_3_)_2_·6H_2_O and 0.6 g of C_9_H_6_O_6_ each were dissolved in 30 mL of ethanol by constant stirring for 30 min. Subsequently, both solutions were mixed together and continuously stirred for another 60 min. Then, the mixture was transferred to a 75 mL Teflon-lined stainless-steel autoclave at a rate of approximately 5 °C per minute. The reaction lasted for 14 h at 130 °C. Afterwards, the autoclave was cooled down to room temperature. The resultant milky crystal precipitate of Zn-BTC MOF was centrifuged, washed several times with fresh ethanol and deionized water, and dried in a vacuum oven at 80 °C for 12 h. The yield of the Zn-BTC MOF prepared at this stage, compared to the metal salt used, was about 61.1%.

### Composite electrode fabrication

Composite electrode fabrication consists of two stages as shown in Fig. S2: (1) the preparation of the electrode ink and, (2) coating the resultant ink on a current collector. Ink preparation is the key step, so that it is essential for the resulting ink to be homogeneous. In preparing the ink, six different electrode compositions (i.e., E1, E2, E3, E4, E5, and E6), including three components (i.e., AC, PVDF, and Zn-BTC MOF), were investigated, as observed in Table S1. For the preparation of each composite electrode, AC, PVDF, and Zn-BTC MOF were weighted according to specified composition formula as indicated in Table S1. Then NMP solvent was added to each composition, and they were immersed in an ultrasonic bath for 20 min. A completely homogeneous ink was prepared by placing it on an electromagnetic stirrer at ambient temperature for at least 12 h, and consequently, cyclic voltammetry (CV) and electrochemical impedance spectroscopy (EIS) tests were conducted in a three-electrode cell. The electrode with best performance in these electrochemical tests was then selected for further characterization and desalination tests.

The specific capacitance and overall electrochemical resistance were measured using CV and EIS tests, respectively^25,54^. For this purpose, CVs were conducted in 1.0 M NaCl at a rate of 5.0 mV s^−1^ [versus Ag/AgCl] for potential range of − 0.5 up to + 0.5 V. EIS was also conducted for a frequency range of 700.0 kHz to 1.0 mHz, with the alternating potential amplitude being 10.0 mV around the open circuit potential. Each of the prepared inks was coated on a glassy carbon (2.0 mm diameter) as a working electrode. The counter electrode was a 3.0 cm^2^ rectangular platinum (Pt) plate, and the reference electrode was an Ag/AgCl electrode in saturated KCl.

The specific capacitance values (C) (F g^−1^) were determined using the I–V curve according to Eq. (1) ^55^:

1 $$C=\frac{S}{\Delta Vm\vartheta }$$

where *S* is the area surrounded by the CV curve, $\Delta V$ is the potential window (V), *m* is the mass of active material on electrodes (g), and $\vartheta$ is the potential scan rate (V s^−1^).

The SRC (mg g^−1^), mean salt removal capacity (MSRC) (mg g^−1^ min^−1^), and salt removal efficiency (SRE) (%) were calculated based on the initial and final concentrations of the feed solution using Eqs. (2), (3), and (4)^56,57^:

2 $$SRC=\frac{\left({C}_{f}-{C}_{0}\right)\times V}{m}$$

3 $$MSRC=\frac{SRC}{t}$$

4 $$SRE=\frac{{C}_{f}-{C}_{0}}{{C}_{0}}\times 100$$

where *C*_*f*_, *C*_*0*_, *V*, *m,* and* t* are the final concentrations (mg L^−1^), initial concentrations (mg L^−1^), reservoir volume (mL), electrode mass (g), and time (min), respectively.

After selecting the most suitable composition of electrode in terms of electrochemical performance by CV and EIS tests, it is necessary to test the selected electrode in a CDI cell to evaluate its desalination performance. The composite electrode fabrication process for desalination tests was as follows. First, for each desalination test, the selected electrode composition (as the active layer of the electrode) was coated with a soft brush onto two circular pieces of carbon cloth (each 4.0 cm in diameter) placed on the surface of two graphite sheets (each 8.0 cm in diameter) as anode and cathode; then the electrodes were completely dried in three steps by a vacuum oven; at 60 °C for 3 h, at 80 °C for 2 h and at 100 °C for 1 h. The electrodes were then removed from the oven and allowed to cool to ambient temperature. The electrodes were rinsed with deionized water to remove contaminants. Finally, the electrodes were placed in a vacuum oven at 100 °C for 2 h to dry completely. At last, the total dried mass of the active layer on each composite electrode was 0.11 g with 260 µm in thickness. The CDI tests were conducted in a batch setup at ambient temperature using a 50.0 mL NaCl feed solution with an initial concentration of 100.0 mg L^−1^ (with an initial electrical conductivity of 253.4 µS cm^−1^), which is regarded as brackish water. The NaCl solution conductivity was monitored with a conductivity meter during the test. The relationship between conductivity and NaCl concentration was obtained by preparing a calibration curve before the experiments.

### CDI experimental setup

**Figure 1:**
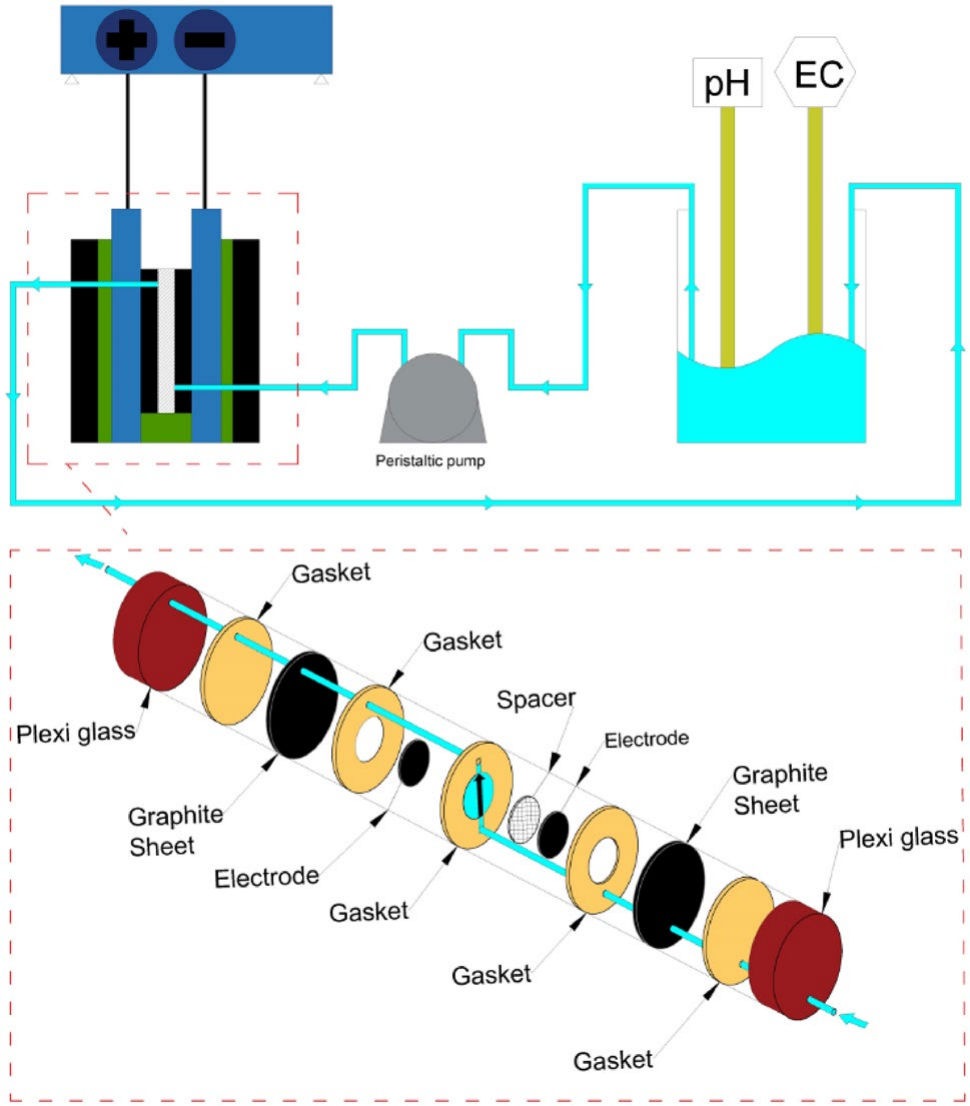
Schematic diagram of CDI cell used in this work.

CDI experiments were performed with a batch-mode setup. It contained a feed solution reservoir, a peristaltic pump (Lab 2015, Shenchen Co., China), a Galvanostat/Potentiostat device (SP-150, Bio-Logic Science Instruments SAS, France), a conductivity meter (EC-470 L, ISTEK Co., Korea), a pH meter (P25, ISTEK Co., Korea), and a lab-made CDI unit cell. To investigate the performance of the electrodes, a CDI device (Fig. 1) was constructed. This device consisted of two circular sheets of plexiglass for encasement, two composite electrodes containing active material coated on carbon cloth fixed on a circular graphite sheet as current collectors, and separated by a nylon mesh spacer. Additionally, several silicone rubber gaskets were used for sealing.

### Physicochemical/electrochemical characterizations

To evaluate Zn-BTC MOF crystal formation, X-ray diffraction (XRD) test was performed at the 2Θ angle range from 5 up to 90 degrees, using the Philips Xpert device (Netherlands) and Cu-Kalpha radiation source. In order to investigate the chemical structure of H_3_BTC, Zn-BTC MOF, AC, and fabricated electrodes, Fourier transform infrared (FTIR) tests were carried out using the Thermo Electron Scientific Instruments LLC device (USA) in the spectral range of 400–4000 cm^−1^. To study the morphology of the Zn-BTC MOF and the composite active material of electrodes, field emission scanning electron microscopy (FESEM) images with magnifications of 1, 5, and 20 µm were taken with a MIRA3 TESCAN device (Czech Republic), enabling a comprehensive analysis of their structural characteristics. Energy dispersive X-ray spectroscopy (EDS) and Elemental Mapping tests were conducted with a MIRA3 TESCAN device (Czech Republic) to further confirm the elemental composition and distribution within the materials. The particle size distribution of Zn-BTC MOF was also analyzed and estimated using the ImageJ software. The SSA and mean pore diameter of AC and Zn-BTC MOF were calculated based on the adsorption–desorption isotherms of nitrogen gas at liquid nitrogen temperature by the Brunauer–Emmett–Teller (BET) method using a Belsorp system (BEL Japan, Inc.). Contact angle (CA) tests have been applied to investigate the hydrophilicity and wettability of the Zn-BTC MOF and electrodes with an Integrated Exploitation System of Laboratory Equipment (IUST, Iran). A SP-150 Galvanostat/Potentiostat device (Bio-Logic Science Instruments SAS, France) was used as a potential supplier in CV, EIS, and desalination tests.

## Results and discussion

### Zn-BTC MOF physicochemical characterization

The XRD test was performed to confirm the construction of the Zn-BTC MOF. It is necessary to match the spectrum obtained from the as-prepared MOF with the spectrum obtained from samples reported in previous studies^48,58,59^. Figure S3a illustrates the XRD spectrum of the Zn-BTC MOF synthesized in this work and the XRD spectrum of the samples synthesized by Osman et al.^59^. According to Fig. S3a, a highly intense peak at 2Θ = 10°, and some minor peaks at 2Θ = 15.64°, 17.72°, and 26.16° are observed, confirming the successful construction of the Zn-BTC MOF^48,58,59^.

FTIR is another test employed for investigating the Zn-BTC MOF structure, considering that the bonds in the Zn-BTC MOF are formed by H_3_BTC organic ligand molecules^60,61^. The FTIR spectrum of the Zn-BTC MOF and the spectrum of the H_3_BTC organic ligand were measured, as shown in Fig. S3b, which are described in supplementary information.

The morphology of Zn-BTC MOF particles was investigated using FESEM images. Figure S4a shows images at 1, 5 and 20 μm magnifications. The shapes of the Zn-BTC MOF particles are spherical and polyhedral. The existence of two different shapes (spherical and polyhedral) with different sizes for the Zn-BTC MOF particles can be caused by small variations in temperature during the synthesizing stage in the autoclave^50,62,63^.

Particle size distribution has been obtained from FESEM images using ImageJ software. Figure S4b shows that particles with a diameter between 30 and 500 nm are most abundant. The presence of nano and micro-particles in composite electrode structure can be influential in two ways. The use of Zn-BTC MOF with nanometer-scale dimensions can improve dispersion and uniformity in the electrode structure and thus enhance the overall stability of structure. Conversely, coarser particles with micrometer-scale dimensions can afford more space between AC particles in the electrode, and consequently leading to better diffusion and greater access of ions to the active sites within the electrode structure^64,65^.

EDS test is used to identify the type and quantity of elements and the elemental mapping test is used to determine the quality of elemental distribution. The EDS result as shown in Fig. S5a, confirms the elemental composition of the Zn-BTC MOF (i.e., C, O, Zn, and N). However, the additional peak observed belongs to aluminum, which is caused by the aluminum surface of the sample holder^63,66^. Also, in Fig. S5b, the elemental distribution of the MOF can be seen, which demonstrates the well distribution of all elements in the structure.

The SSA, pore size distribution, and pore volume of the Zn-BTC MOF were assessed using the BET test, as well as nitrogen adsorption and desorption isotherms. These analyses generated relevant graphs and tables, which are presented in Fig. S6 and Table S2, respectively. According to the adsorption and desorption diagram in Fig. S6a, the adsorption isotherm of this MOF is of the fourth type with a hysteresis loop of the third type^17,67^. This shows non-hard and plate-like meso and micro pores presented in its structure^17,63,67^. According to Fig. S6 and Table S2, an SSA of 34 m^2^ g^−1^, a pore volume of 0.096 cm^3^ g^−1^, and a Mean pore diameter of 11.54 nm were achieved using Burt–Joyner–Holland (BJH) method. It's demonstrated that a mesoporous structure has better performance than macro and microporous structures for adsorbing ions from the feed solution and forming the electrical double layer (EDL)^17,68,69^.

According to Fig. S7, the CA of water with the tablet prepared from Zn-BTC MOF powder is 26.7 degrees, which confirms the high wettability and hydrophilicity of this material^30,69^.

### Electrodes physicochemical/electrochemical characterization

The electrochemical performance [i.e., Specific capacitance (F g^−1^) and Ion charge transfer resistance (Ω)] of the six electrodes with different composition is indicated in Table 1.

**Table 1:** Results of CV and EIS tests of the electrodes.

No.	AC (wt%)	PVDF (wt%)	Zn-BTC MOF (wt%)	Specific capacitance (F g^−1^)	Ion charge transfer resistance (Ω)
E1	92	8	0	60	7.5
E2	90	8	2	74	6.3
E3	88	8	4	83	5.4
E4	86	8	6	88	4.9
E5	84	8	8	78	5.1
E6	82	8	10	67	5.6

The adsorption potential of an electrode strongly hinges on its capacity to hold and retain ions within its structure^19,70^. Thus, electrodes exhibiting higher specific capacitance values in the CV test are expected to demonstrate superior adsorption performance^19,71^. According to Table 1, the addition of up to 10% of the Zn-BTC MOF results in a maximum increment of 46.7% in the specific capacitance of electrodes from E2 to E6 compared to that of E1. A relatively sharp increase in ion charge transfer resistance was observed for MOF loadings greater than 6%, probably due to decreased overall electrical conductivity and active surface of the electrode^70,72,73^. As a result, the highest specific capacitance and minimum ion charge transfer resistance were obtained using the E4 electrode, which contained 6% of the Zn-BTC MOF. This outcome is likely related to the high hydrophilicity of the Zn-BTC MOF as well as the proper pore size distribution of the electrodes^69,74^. The overall behavior of CV and EIS test results indicates that the addition of a small quantity of Zn-BTC MOF and its optimization with other materials in the composition of electrodes lead to the enhancement of synergistically characteristic of electrode performance, which greatly impacts the specific capacitance and charge transfer kinetics of composite electrodes^70,74,75^.

**Figure 2:**
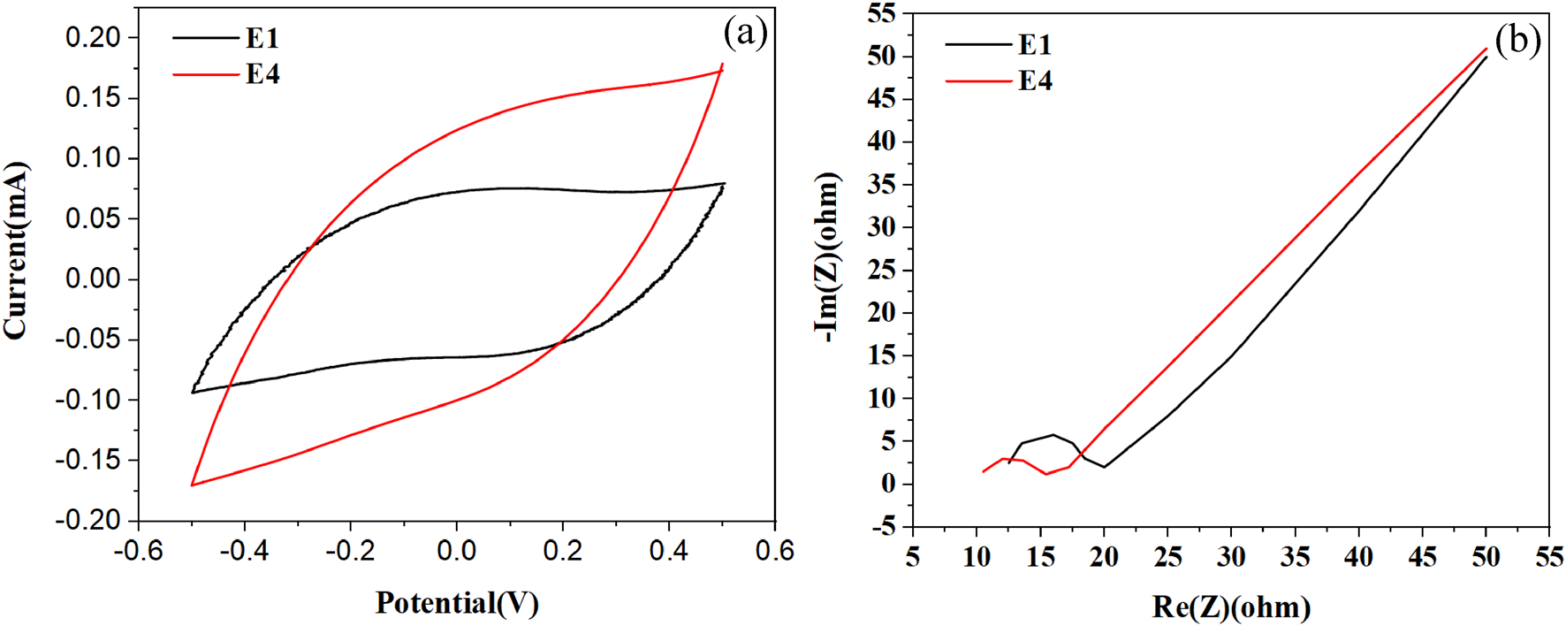
(**a**) The CV; and (**b**) EIS diagrams of E1 and E4 electrodes.

Therefore, further examination was conducted only on E1 and E4 electrodes to better reveal the superior performance of E4 as most appropriate electrode in CDI process. In the first step, the graphs obtained from CV and EIS characterizations were analyzed for E1 and E4 electrodes. Figure 2a shows CV test behavior for E1 and E4 electrodes. The CV curves of the electrodes have a quasi-rectangular shape and do not have peaks caused by Faradaic reactions, which confirms the capacitive behavior of the electrodes due to the formation of the EDL^17,76^. As a result, a greater surface area of the closed loop corresponds to a higher ion charge adsorption capacity of the electrode^17,25^. Also, The presence of CV curves signifies the reversible nature of the capacitive adsorption performance of the electrodes^17,25,77^. The elevation of the current slope observed at the initial and final stages of the CV curve for electrode E4, in contrast to E1, reflects an enhanced hydrophilicity and reduced electrical resistance of E4 compared to E1^69,76–78^.

Figure 2b shows the Nyquist curves obtained from the EIS test of E1 and E4 electrodes. As mentioned earlier, the EIS test indicates electrochemical resistances, especially ion charge transfer resistance in electrodes. In the EIS diagram, the vertical axis, which is imaginary resistance, is related to the capacitive resistance of the electrode, and the horizontal axis, which is the real resistance, is related to the electrical resistance of the solution, the charge transfer resistance in the electrode, and the ion diffusion resistance in the electrode^17,25,72^. The first intersection point of the curve with the horizontal axis indicates the electrical resistance of the electrolyte solution^17,76^. Also, the semicircle range at high-frequency values in the plot reflects the contact resistance of the electrode/electrolyte, which affects the efficacy of ion transfer^25,74,76^. Both electrodes exhibit similar shapes and trends in their respective plots. Upon the introduction of Zn-BTC MOF into the E4 electrode, a smaller half-circle is observed as compared to the E1 electrode, indicative of lower ion charge transfer resistance within the electrode structure and potentially better diffusion of ions. However, the slope in the low-frequency region of the plot reflects the rate of ion diffusion, which is found to be nearly equivalent for both electrodes^17,25,73,74,76^.

**Figure 3:**
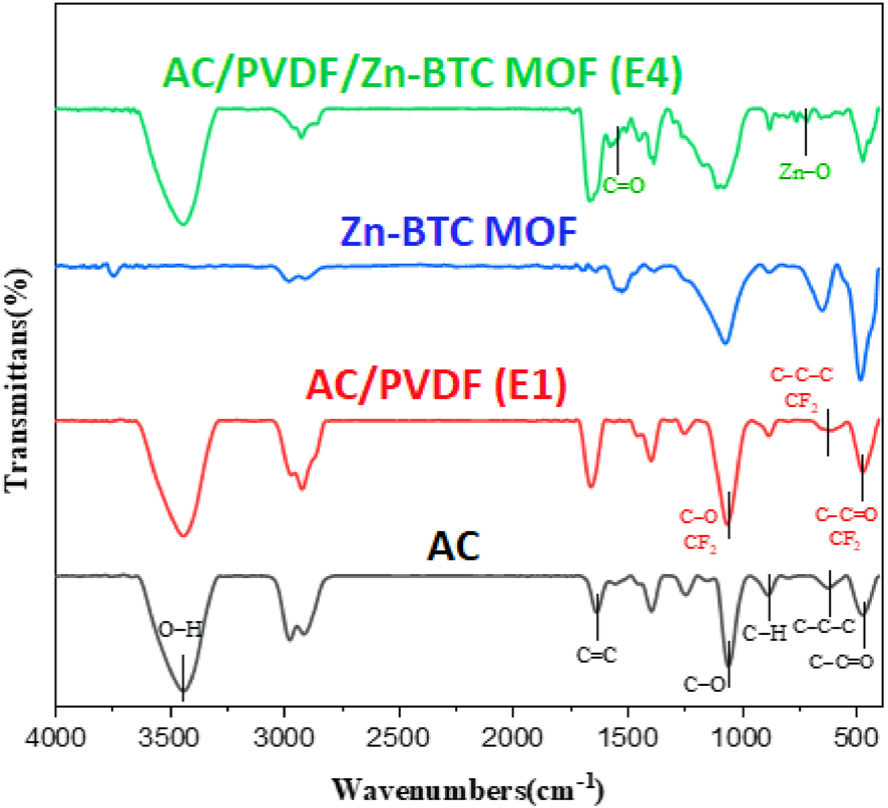
FTIR spectrum of AC, E1 electrode, Zn-BTC MOF and E4 electrode.

FTIR results of AC, Zn-BTC MOF, E1, and E4 electrodes are shown in Fig. 3. According to that, the AC spectrum shows peaks at 1060 cm^−1^, 1635 cm^−1^, and 2820 cm^−1^ to 3633 cm^−1^ related to C–O, C=C, and O–H bonds, respectively^17^. Additionally, peaks at 474 cm^−1^, 624 cm^−1^, and 890 cm^−1^ are caused by C–C=O, C–C–C, and C–H bonds in the AC structure^79^. The spectrum of the E1 electrode is very similar to AC's spectrum, and due to the small amounts of PVDF and the overlap of a number of PVDF and activated carbon indicator peaks, no apparent difference is observed in the spectrum of the E1 electrode and AC. In general, the peaks at 470 cm^−1^, 621 cm^−1^, and 1064 cm^−1^ in the E1 electrode spectrum, in addition to being related to C–C=O, C–C–C, and C–O bonds, can also indicate the presence of CF_2_ bonds^79,80^. Also, the peaks at 1458 cm^−1^ and 2970 cm^−1^ confirm the presence of CH_2_ bonds^79^. In the E4 electrode spectrum, the effect of increasing the Zn-BTC MOF on the mixture of AC and PVDF is observed. According to this spectrum, the weak peak at 717 cm^−1^ is due to the presence of a Zn–O bond^48,81^, and the peak ranging from 1480 to 1596 cm^−1^ equally belongs to the carboxyl group of the benzene ring due to the presence of a C=O bond^46,48,81^.

The FESEM images of the E1 and E4 electrodes are shown in Fig. S8a and b, respectively, at three magnifications of 1, 5, and 20 μm. The good pore distribution and particle dispersity of compositions in the E4 electrode can be clearly observed, revealing the effect of MOF on the structure of the electrode, as compared to the E1 electrode.

The EDS results of the E1 and E4 electrodes are shown in Fig. S9a and b, respectively, as well as the elemental mapping images of both electrodes in Figs. S10 and S11, which are described in supplementary information.

**Figure 4:**
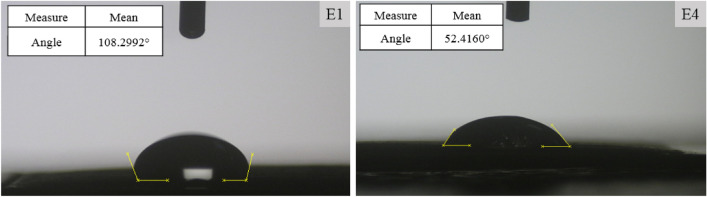
CA of E1 and E4 electrodes.

Enhancing the hydrophilicity and wettability of the electrode surface can promote more efficient diffusion of ions within the electrode matrix^17,30^. Therefore, more pores participate in the ion adsorption process^17^. In addition, more active electrode surface is available to ions^73^. In Fig. 4, the CA of water with E1 and E4 electrodes can be seen. The CA of E1 and E4 electrodes is 108.3 and 52.4 degrees, respectively. PVDF binder and AC are both hydrophobic materials that generally make electrodes more hydrophobic^17,29^. Given the prominently high hydrophilicity exhibited by the Zn-BTC MOF, the observed rise in hydrophilicity of the E4 compared to the E1 electrode is consistent with prior studies^29,30,73^. The magnitude of the observed increment in hydrophilicity can result in a concomitant elevation in the total quantity and rate of ionic diffusion into the porous structure of the electrode^17,69,73^. Consequently, a broader and more stable EDL is established on the active surface of the electrode^17,29,73^.

According to the adsorption and desorption diagram (Fig. S12a), the adsorption isotherm of AC is of the fourth type with a hysteresis loop of the third type, indicating the presence of non-hard, plate-like meso and macropores in its structure^22,47,54^. Additionally, Fig. S12 and Table S3 show an SSA of 723 m^2^ g^−1^, a pore volume of 0.364 cm^3^ g^−1^, and a Mean pore diameter of 3.10 nm that were achieved using BJH method. Materials with mesoporous structures exhibit superior performance compared to those with macro and microporous structures in terms of ion adsorption and the formation of an EDL within the material, effectively accommodating ions and facilitating ion diffusion^17,68,69^.

### Electrodes desalination performance

In the CDI cell, the desalination process was executed using three different electrode arrangements: SymE1 (symmetric arrangement, with E1 used as both anode and cathode), SymE4 (symmetric arrangement, with E4 used as both anode and cathode), and Asym (asymmetric arrangement, with E4 used as anode and E1 as cathode). These arrangements were selected for two main reasons: (1) to study and compare the effect of adding Zn-BTC MOF to electrodes in a symmetric arrangement on the increase in SRC, and (2) to investigate the potential impact of the positive charge density of Zn-BTC MOF in the anode on both the electrical field force induced by externally applied voltage and the interaction forces of ions with electrodes in an asymmetric arrangement. Each desalination test was repeated three times, from a NaCl feed solution with an initial concentration of 100.0 mg L^−1^ and an initial electrical conductivity of 253.4 µS cm^−1^. It is important to determine the appropriate conditions for the CDI process to achieve the best performance. Therefore, a suitable applied potential difference was determined. Insufficient applied voltage leads to a reduced formation of a suitable EDL, causing a decrease in the adsorption capacity of the electrode^17,70^. Excessive applied voltage can trigger Faradaic reactions or electrolysis of water, compromising the accuracy and stability of the electrode–electrolyte system^17,82,83^. Therefore, determining the optimal voltage for CDI cells is of particular importance.

For the SymE1 arrangement, the results of desalination of a NaCl feed solution at voltages of 1.2 V and 1.6 V, and a flow rate of 20 mL min^−1^, are depicted in Fig. S13a. It should be noted that due to the intense changes in the pH of the solution at a voltage of 2.0 V and the occurrence of Faradaic reactions^17,82,83^, the deionization process was stopped at this voltage, and therefore its results are not presented. According to Fig. S13a and some pre-tests in different voltages in all three different arrangements of electrodes, the best voltage was determined to be 1.6 V. As can be seen at the voltage of 1.6 V, the electrical conductivity of the feed solution has decreased to a greater extent in a period of 30 min, which means more desalination. The SRC at 1.2 and 1.6 V was equal to 2.4 and 5.2 mg g^−1^, respectively, while the SRE was measured at 8.4% and 18.1%, respectively. This clearly indicates the direct effect of the electrical field force induced by the applied voltage on the amount of salt adsorption by the CDI cell^25^. Furthermore, the absence of gas bubbles and lack of intense pH changes suggests that Faradaic reactions or water electrolysis did not occur^17,82,83^.

In another test to determine the appropriate flow rate of feed solution at 1.6 V, the SRC at flow rates of 10, 20, and 30 mL min^−1^ resulting in corresponding values of 4.7, 5.2, and 4.1 mg g^−1^, respectively, as shown in Fig. S13b. The corresponding SRE values for these flow rates were found to be 16.3%, 18.1%, and 14.3%, respectively. Based on the results from Fig. S13b and pre-tests using different flow rates and electrode arrangements, the optimal flow rate was determined to be 20 mL min^−1^. This outcome can be attributed to the effective diffusion of ions and the establishment of a stable EDL in the porous electrode structure, facilitated by adequate time^84,85^. Therefore, the assessment of desalination process were performed at the voltage of 1.6 V and the flow rate of 20 mL min^−1^.

**Figure 5:**
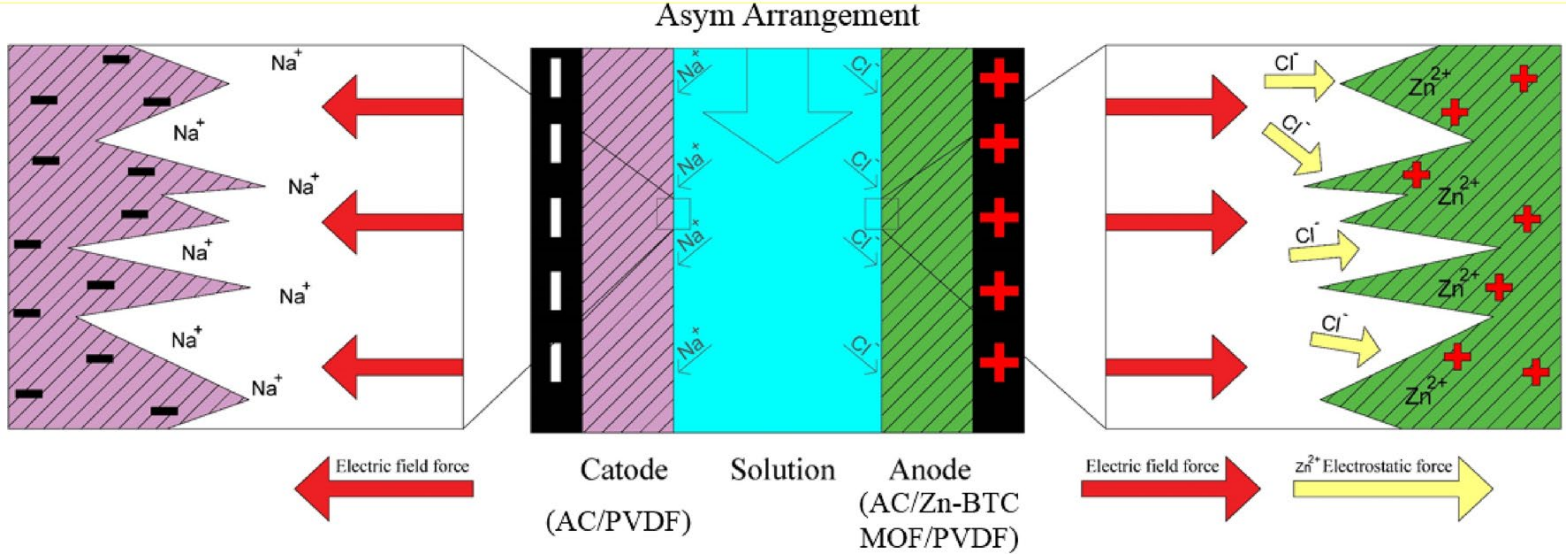
Schematic mechanism involved in the Asym arrangement in CDI system.

The results of desalination in all three arrangements (SymE1, SymE4, and Asym) are shown in Fig. S13c. The SRC within 30 min of the desalination process for SymE1, SymE4, and Asym arrangements is equal to 5.2, 6.0, and 6.3 mg g^−1^, respectively, which are equivalent to 18.1%, 20.8%, and 21.9% of SRE, respectively. As expected, the SymE4 has more desalination than the SymE1 arrangement. The high hydrophilicity of the Zn-BTC MOF, coupled with the greater specific capacitance and lower ion charge transfer resistance of the E4 electrode compared to E1, results in faster and more efficient ion diffusion and a more stable formation of the EDL within the electrode structure^17,73^. Additionally, the Asym arrangement exhibits more desalination efficiency than the SymE4 arrangement. The reason behind this phenomenon refers to the electrostatic interactions from the electrical field induced by the applied voltage on the electrodes and the charge of zinc ions (Zn^2+^) present within the Zn-BTC MOF structure^29,60^. The presence of Zn^2+^ ions in MOF structure creates positively charged sites that have a higher charge density than –COO– groups in the structure^29,53,86^. The anode, being the positively charged electrode, exhibits a distinct behavior because of the incorporation of Zn-BTC MOF along with the electric force effect of the externally applied voltage field^29^. The presence of Zn^2+^ ions within the MOF structure exerts an attractive electrostatic force on the anions, thereby leading to enhanced attraction and separation of a larger number of anions from the passing solution comprising Na^+^ and Cl^−^ ions within the CDI cell^85^. This consequently leads to the formation of a stable EDL within the porous structure of the electrode^53,60,87,88^. The schematic mechanism involved in the Asym arrangement in the CDI system is depicted in Fig. 5.

Furthermore, it can be inferred that the inclusion of Zn-BTC MOF within the cathode material of the SymE4 arrangement improves the electrode specific capacitance and concurrently reduces the ion diffusion resistance^86,89^. However, the presence of Zn^2+^ ion sites within the cathode structure could potentially decreases its performance due to the consequent repulsion between Na^+^ and Zn^2+^ cations. Consequently, a decrease in cation adsorption can lower the performance of the SymE4 in comparison to the Asym arrangement^29,53,89^. The results of SRC and SRE of all three arrangements, considering the possible error for each arrangement, are shown in Fig. S13d and e, respectively.

**Figure 6:**
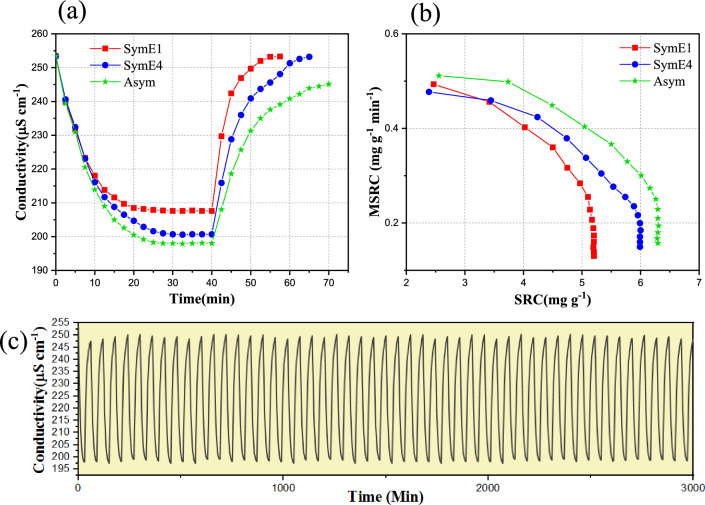
(**a**) The results of one cycle of adsorption and desorption process for all the three arrangements of electrodes. (**b**) CDI Ragone plots of all the three arrangements of electrodes. (**c**) The adsorption and desorption cycling stability test of Asym arrangement of electrodes for 50 cycles. Experimental conditions: a voltage of 1.6 V and a flow rate of 20 mL min^−1^.

The results of a complete CDI process cycle (adsorption and desorption) are given in Fig. 6a. The desorption stage that removes ions from the electrodes (electrode regeneration) is carried out at 0.0 V and a flow rate of 20 mL min^−1^. It can be seen that in the SymE1 arrangement, the electrodes are fully regenerated faster. After 15 min from the desorption stage, the feed solution electrical conductivity returns to its initial value. In the SymE4 arrangement, it takes 25 min for the electrodes to be completely regenerated and for the electrical conductivity of the feed solution to return to its initial value. This may be due to the increased adsorption of ions during the adsorption stage, leading to a prolonged time interval for their subsequent removal^29,60,87^. Additionally, the proposed arrangement exhibits a higher degree of stability of EDL compared to that of the SymE1 arrangement^29,86,89^.

In the Asym arrangement, even after 30 min of the desorption stage, the electrodes have not been fully regenerated, and the electrical conductivity of the feed solution has not reached the initial value. This phenomenon can arise from several reasons. Firstly, the enhanced ionic adsorption in the adsorption stage can lead to a subsequent elongation in the ion removal time^29,60,87^. Secondly, the formation of a more stable EDL in the proposed arrangement outperforms that of both the SymE1 and SymE4 arrangements^29,86,89^. Last but not least, the anode comprises the Zn-BTC MOF in which the positive charge density is comparatively higher than the negative charge density^53,86,87^. The anions that accumulate in the EDL of the anode tend to retain their position even after the applied voltage is discontinued. Since the performance and efficiency of the anode and cathode electrodes during deionization processes are interconnected, the behavior of each electrode significantly affects the other^29,89^. This effect is triggered for the cations adsorbed at the cathode as well. Consequently, both SymE1 and SymE4, showed the lower ion removal efficiency and electrode regeneration^29,60,86–88^.

Figure 6b shows CDI Ragone plots for the three arrangements of electrodes. The CDI Ragone plots of both the SymE4 and Asym arrangements shift towards the upper right region compared to SymE1, indicating that they have a higher desalination capacity and desalination rate, possibly due to their increased accessible surface area and mesopores, as well as improved hydrophilicity^29,57,90^. As mentioned before, because of the effects of charge density in the anode and Zn^2+^ electrostatic force of the Asym arrangement, which favor ion diffusion in the pores of the electrode matrix, it demonstrates both a higher desalination capacity and desalination rate compared to the other two arrangements. This indicates the effect of Zn-BTC MOF on capacitive behavior and ion charge transfer kinetics^29,57,75,89^. Figure 6c shows the cyclic adsorption/desorption experiments of the representative Asym arrangement of electrodes in a NaCl feed solution with a starting concentration of 100.0 mg L^−1^. It is noted that the electrodes showed approximately a 2.9% decay in SRC after 50 cycles, proving its good cycling performance.

Table 2 provides the details of the desalination process for all the three arrangements. The process was conducted at a voltage of 1.6 V and a flow rate of 20 mL min^−1^. The initial feed solution contained 100.0 mg L^−1^ NaCl. The details are provided for one cycle of the process. The table demonstrates that incorporating a small quantity of Zn-BTC MOF into composite electrodes, in combination with an asymmetric electrode arrangement, significantly increases SRC. This is evident from the 15.3% and 21.1% increase in SRC for SymE4 and Asym arrangements, respectively, when compared to symE1.

**Table 2:** Details of the desalination process in all the three arrangements.

Arrangement	Mass of electrode active layer (g)	Voltage (V)	Flow rate (mL min^−1^)	NaCl Initial feed concentration (mg L^−1^)	SRC (mg g^−1^)	SRE (%)
SymE1	0.22	1.6	20	100.0	5.2	18.1
SymE4					6.0	20.8
Asym					6.3	21.9

## Conclusion

Incorporation of a small amount of Zn-BTC MOF into the carbon electrodes enhanced the electrochemical and desalination performance. Although the SymE4 and particularly Asym arrangements exhibited weaker performance during the desorption stage, this issue could be resolved either by increasing the flow rate or by applying a reverse voltage for a short duration. Additionally, the electrode mass was periodically measured throughout the experiments, and the lack of any significant mass variation indicated their favorable stability and the absence of noticeable faradaic reactions during the desalination processes.

MOFs, like conventional additives to carbon electrodes, enhances the performance, but MOFs typically exhibit higher SSA and increased active sites. The incorporation of MOF particles with variable sizes ranging from the nanometer to micrometer scale, coupled with the high hydrophilicity of MOFs, enhances MOF particle distribution and uniformity, and also ion diffusion within the electrode structure. This improved dispersion within the AC matrix then promotes better ion accessibility to the electrode porous structure. Zn-BTC MOF exhibits superior desalination performance in asymmetrical arrangement when compared to symmetrical arrangements in CDI systems. This is due to the higher density of positive charge relative to negative charge. Hence, this work demonstrated that composite MOF-incorporated electrodes could be of significant interest for future research aiming at enhancing the performance of CDI systems.

Considering the lack of substantial research on investigating pH changes in adsorption/desorption cycles and their effect on the electrical conductivity of solutions, the importance of studying this aspect in future research is highly significant. Additionally, prioritizing future research should involve examining the optimization conditions of material composition percentages and the selection of metallic nodes and organic ligands for MOF synthesis. It's crucial to address potential challenges associated with scaling up MOF-incorporated electrode production and ensuring its long-term stability in CDI systems. Moreover, investigating the possibility of using the CDI process independently for water desalination or coupling it with other desalination methods like reverse osmosis warrants further exploration.

### Supplementary Information

Supplementary Information.

## Data Availability

The datasets used and/or analysed during the current study available from the corresponding author on reasonable request.
