# Supplementary material for: Enhanced brackish water desalination in capacitive deionization with composite Zn-BTC MOF-incorporated electrodes
Source: Sci Rep. 2024 Jul 1;14:14999. doi: 10.1038/s41598-024-66023-y (PMC11217474; doi:10.1038/s41598-024-66023-y)
Supplement: Supplementary file 1 — Supplementary Information. [file 41598_2024_66023_MOESM1_ESM.docx]

**Supplementary Information**

**Enhanced Brackish Water Desalination in Capacitive Deionization with Composite Zn-BTC MOF-Incorporated Electrodes**

Amirshahriar Ghorbanian^a^, Soosan Rowshanzamir ^a,b^ *, Foad Mehri^a^,

*^a^ Hydrogen & Fuel Cell Research Laboratory, School of Chemical, Petroleum and Gas Engineering, Iran University of Science and Technology, Narmak, Tehran 16846-13114, Iran*

*^b^ Center of Excellence for Membrane Science and Technology, Iran University of Science and Technology, Narmak, Tehran, Iran.*

**^*^**Corresponding *author: Soosan Rowshanzamir, School of Chemical, Petroleum and Gas Engineering, Iran University of Science and Technology, Iran, rowshanzamir@iust.ac.ir*

**Table S1.** Electrodes composition.

| No. | AC (wt%) | PVDF (wt%) | Zn-BTC MOF (wt%) |
| --- | --- | --- | --- |
| E1 | 92 | 8 | 0 |
| E2 | 90 | 8 | 2 |
| E3 | 88 | 8 | 4 |
| E4 | 86 | 8 | 6 |
| E5 | 84 | 8 | 8 |
| E6 | 82 | 8 | 10 |


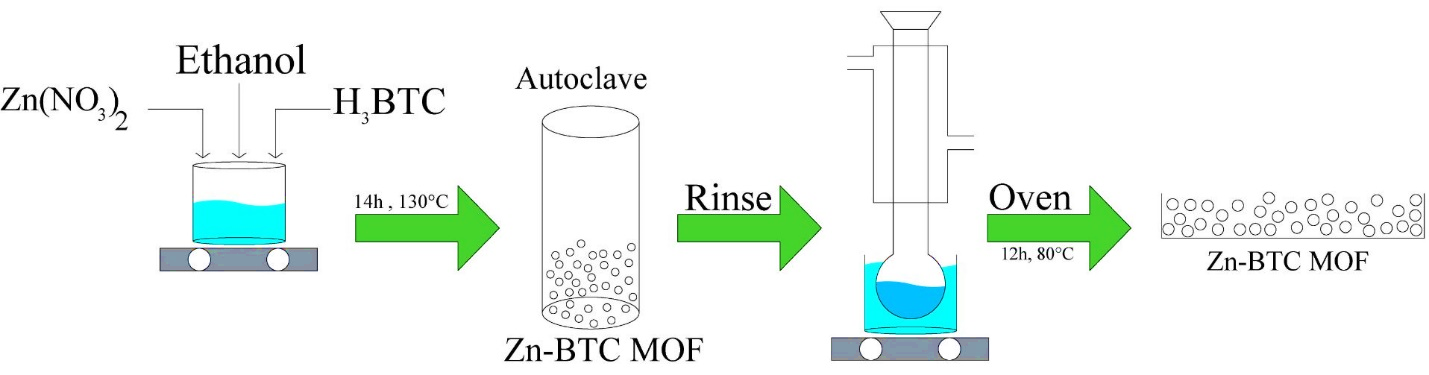


**Figure S1.** Schematic synthetize procedure of Zn-BTC MOF.


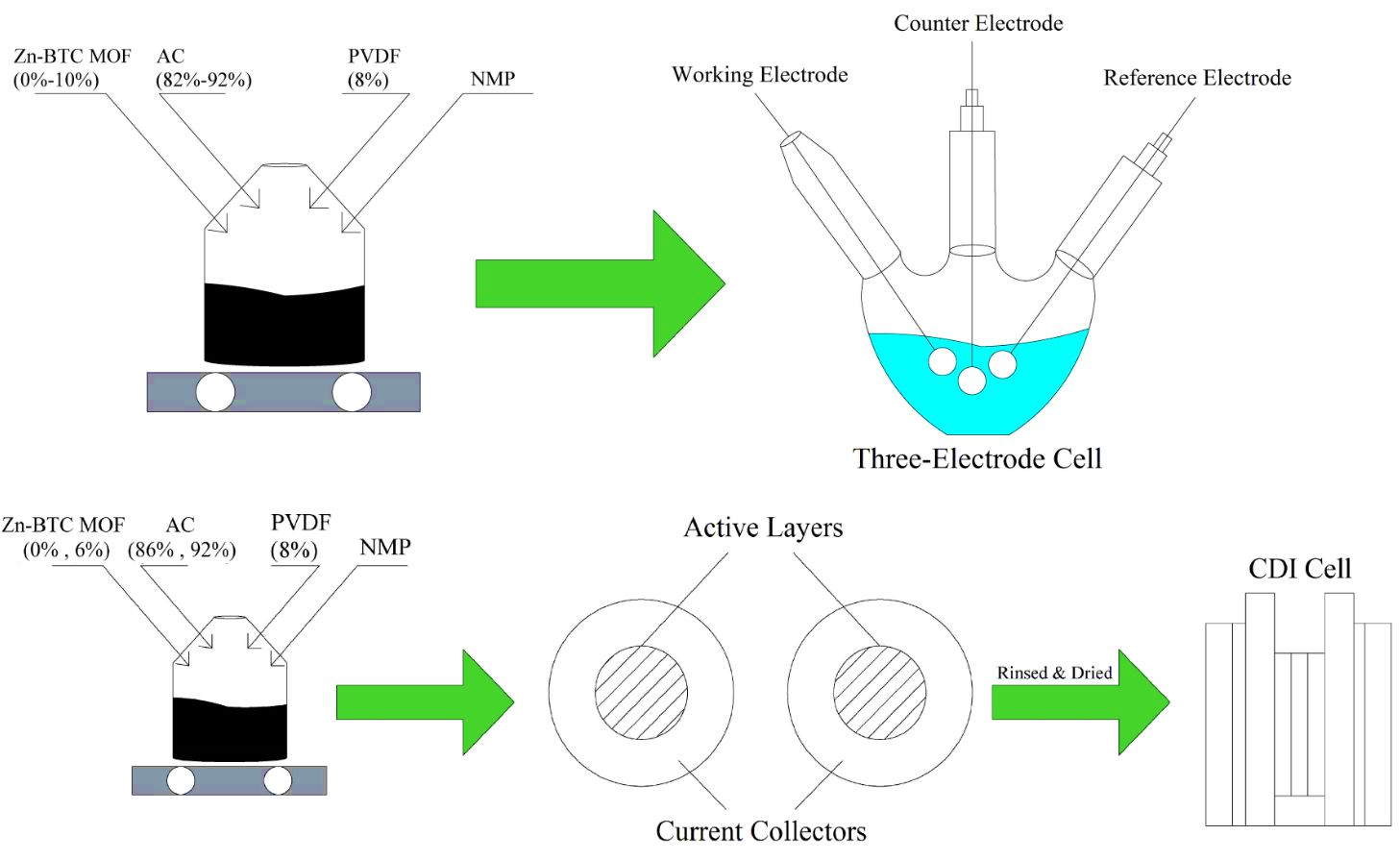


**Figure S2.** Procedure diagram of preparing ink, electrochemical characterization in three-electrode cell, and fabrication of composite electrodes used in CDI cell.

(a)


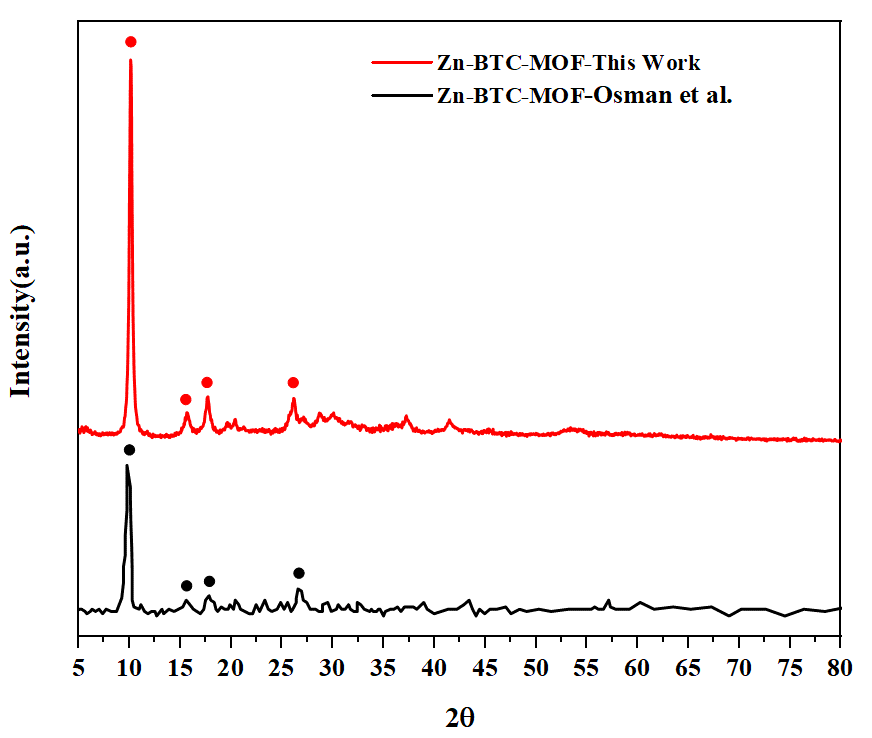


(b)

**Figure S3.** (a) XRD spectrum of the synthesized Zn-BTC MOF in this research and reported in previous studies ^1^; and (b) FTIR spectrum of H_3_BTC organic ligand and the Zn-BTC MOF.

As shown in Figure S3 (b), the peak from 1625 cm^-1^ to 1785 cm^-1^ in the spectrum of H_3_BTC, which corresponds to the C=O bond of the carboxyl group of the benzene ring, has been shifted to the range of 1488 cm^-1^ to 1612 cm^-1^ in the spectrum of the Zn-BTC MOF. This shift is evidence of the formation of a bond between Zn^2+^ and BTC^3-^ ions ^2,3^. The 1608 cm^-1^ peak in the spectrum of H_3_BTC, which is related to the C=C bond of the benzene ring, is also observed in a less intense form at 1484 cm^-1^ in the spectrum of the Zn-BTC MOF ^3^. The very weak peak at 3629 cm^-1^ in the spectrum of H_3_BTC, related to the C−H bond, has been transferred to 3745 cm^-1^ in the spectrum of the Zn-BTC MOF ^3,4^. The peaks in the range of 1376 cm^-1^ to 1477 cm^-1^ in the H_3_BTC spectrum are mainly caused by the O−H bond of the carboxyl group ^2^. Their absence from the Zn-BTC MOF spectrum indicates the involvement of carboxyl groups in bonding with metal ions ^2,3^. The broad peak from 2400 cm^-1^ to 3400 cm^-1^ in the spectrum of H_3_BTC is related to the O−H bond caused by H_2_O molecules. It is a weak peak from 2850 cm^-1^ to 3080 cm^-1^ in the spectrum of the Zn-BTC MOF, mainly caused by the O−H bond of the solvent (ethanol), indicating the relatively complete drying of the Zn-BTC MOF ^2,4^. Additionally, the 648 cm^-1^ peak in the Zn-BTC MOF spectrum is related to the Zn−O bond ^3,5^.

**Figure S4.**  (a) FESEM images of the Zn-BTC MOF at 1, 5 and 20 μm magnifications; and (b) Particle size distribution diagram of the Zn-BTC MOF.


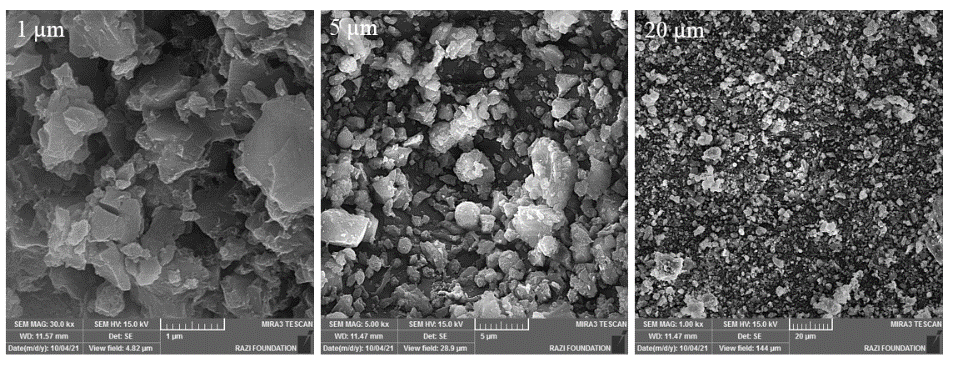

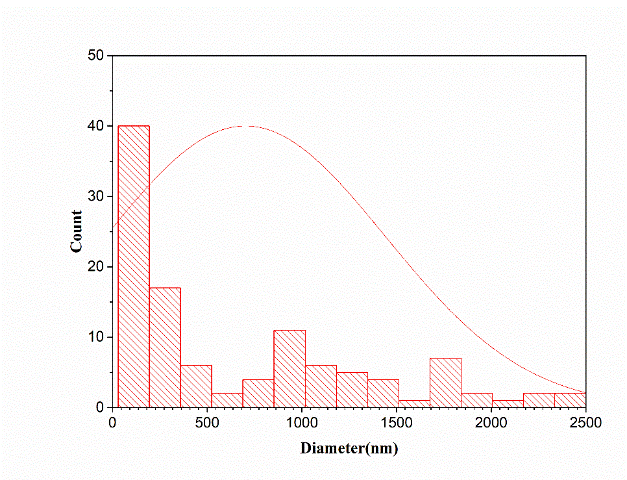


(a)

(b)

(a)


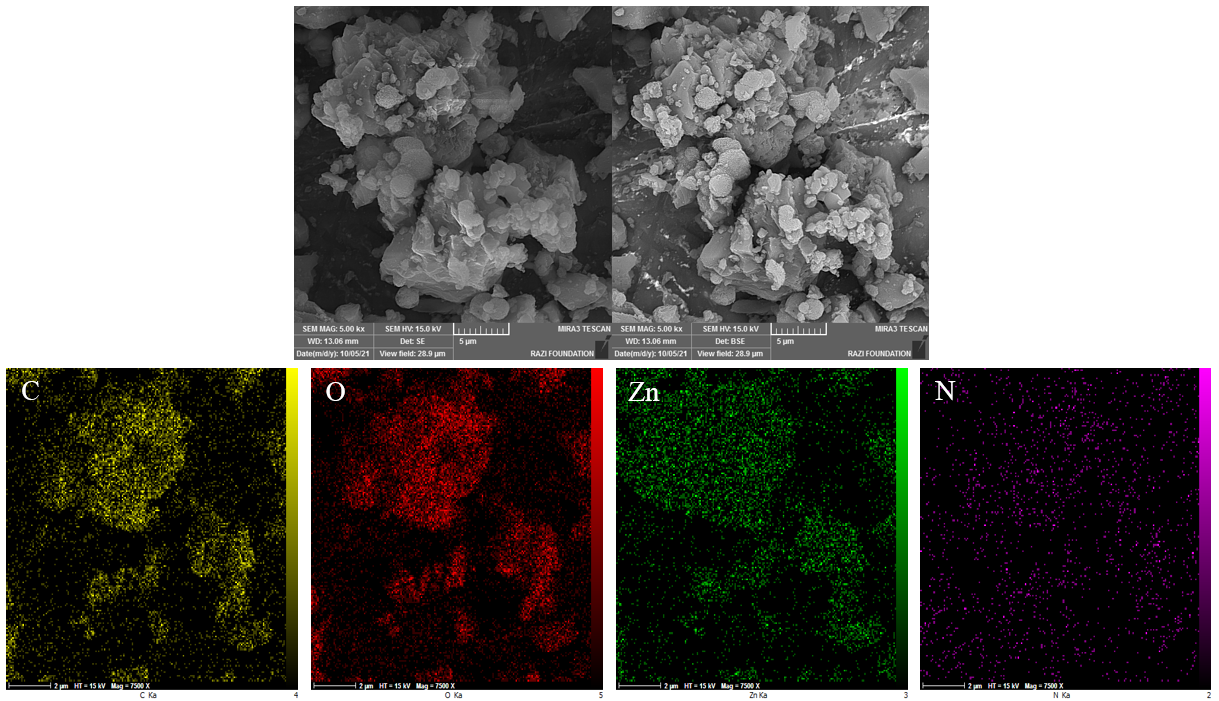

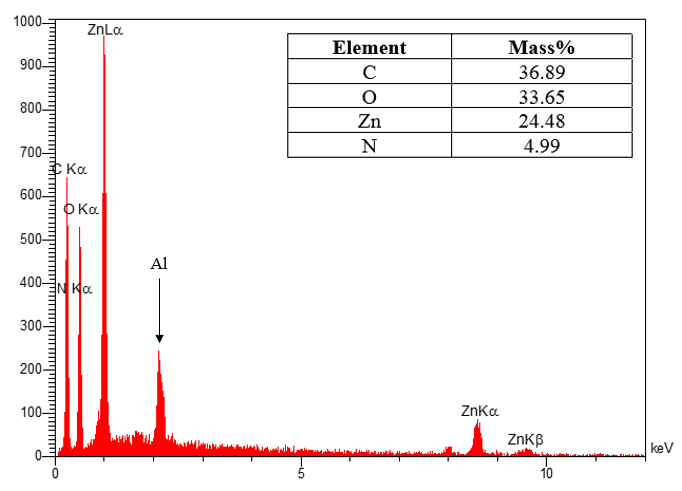


(b)

**Figure S5.** (a) EDS test results for Zn-BTC MOF; and (b) Elemental mapping images of the Zn-BTC MOF.

*
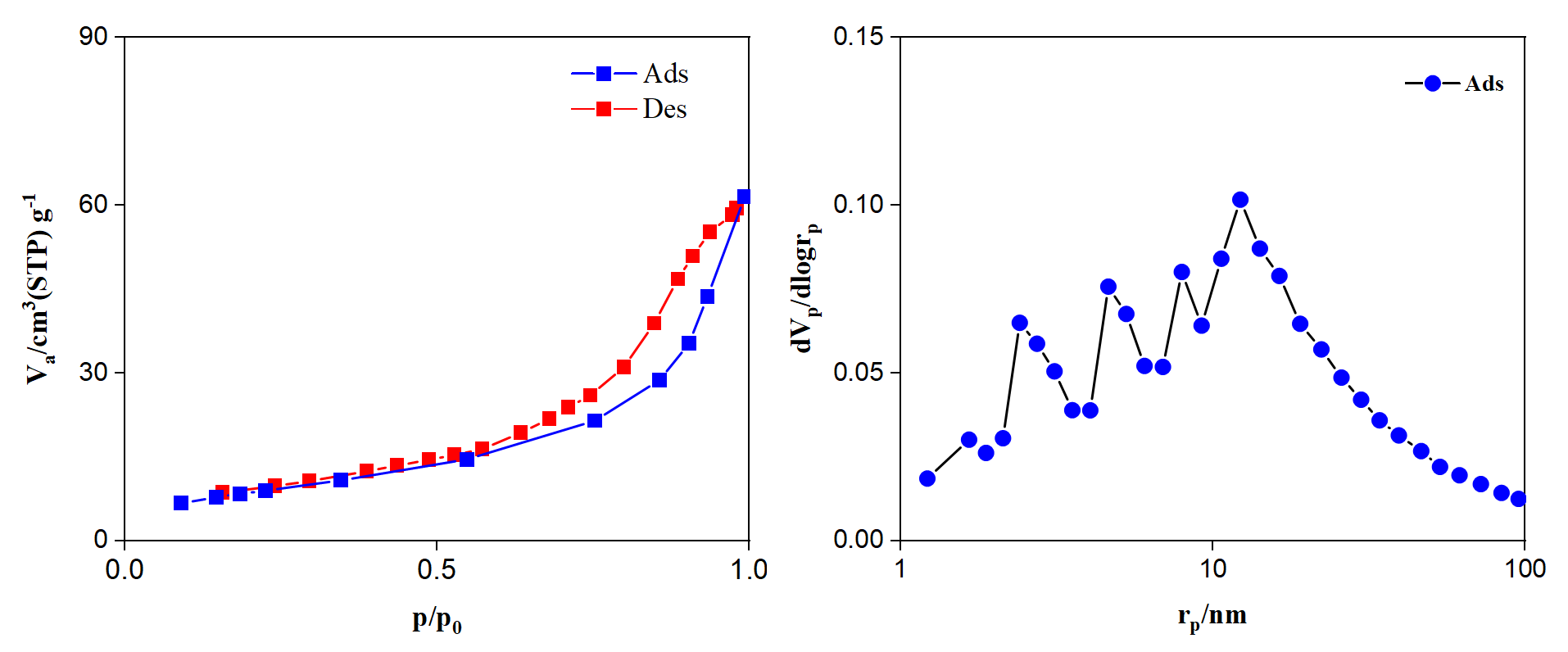
*

(b)

(a)

**Figure S6.** (a) Nitrogen adsorption and desorption diagram; and (b) pore size distribution of Zn-BTC MOF.

**Table S2.** Information from the BET test of the Zn-BTC MOF.

| Material | BET SSA (m^2^ g^-1^) | BJH pores volume (cm^3^ g^-1^) | Mean pore diameter (nm) |
| --- | --- | --- | --- |
| Zn-BTC MOF | 34 | 0.096 | 11.54 |

According to Matzger et al. ^6^ and Yaghi et al. ^7,8^, the BET of the Zn-BTC MOF does not necessarily indicate the real SSA of this MOF. So that, the SSA of synthesized MOF determined by liquid adsorption exhibited higher capacitance than that of obtained by the BET test. The synthesis conditions, along with the shape and size of the MOF pores, may render it unsuitable for gas adsorption due to the limited nitrogen adsorption capacity of the pore walls ^6,7^. Nonetheless, the same MOF framework can exhibit strong adsorption performance in the presence of liquids and demonstrate good stability, especially under aqueous conditions ^7^.


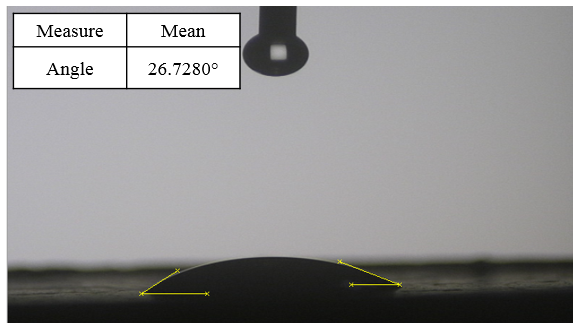


**Figure S7.** CA test of Zn-BTC MOF.

(a)

(b)


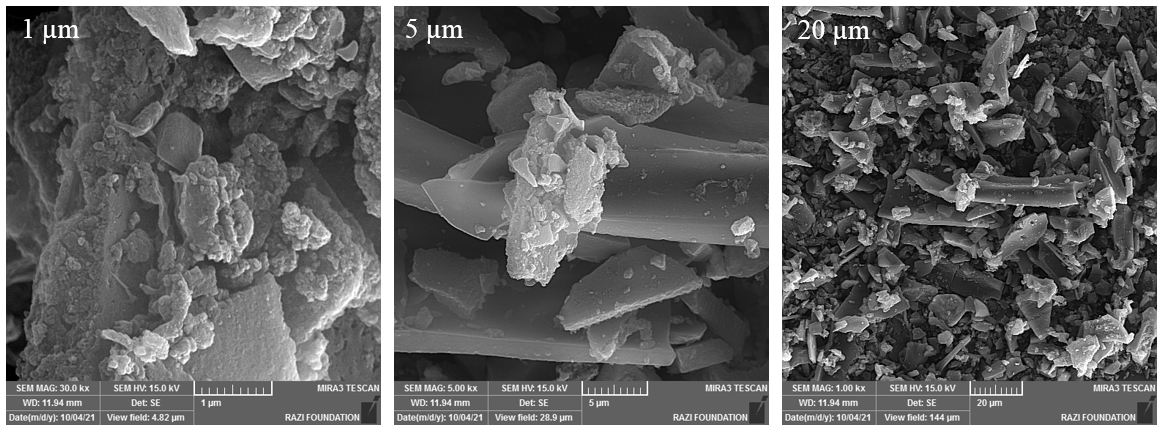

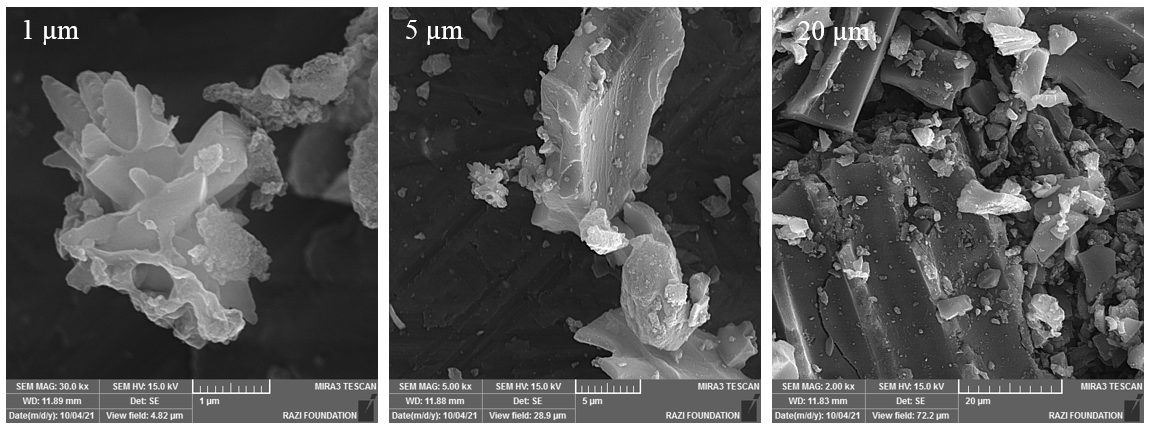


**Figure S8.** (a) FESEM images of E1 electrode; and (b) FESEM images of E4 electrode at magnifications of 1, 5 and 20 μm.


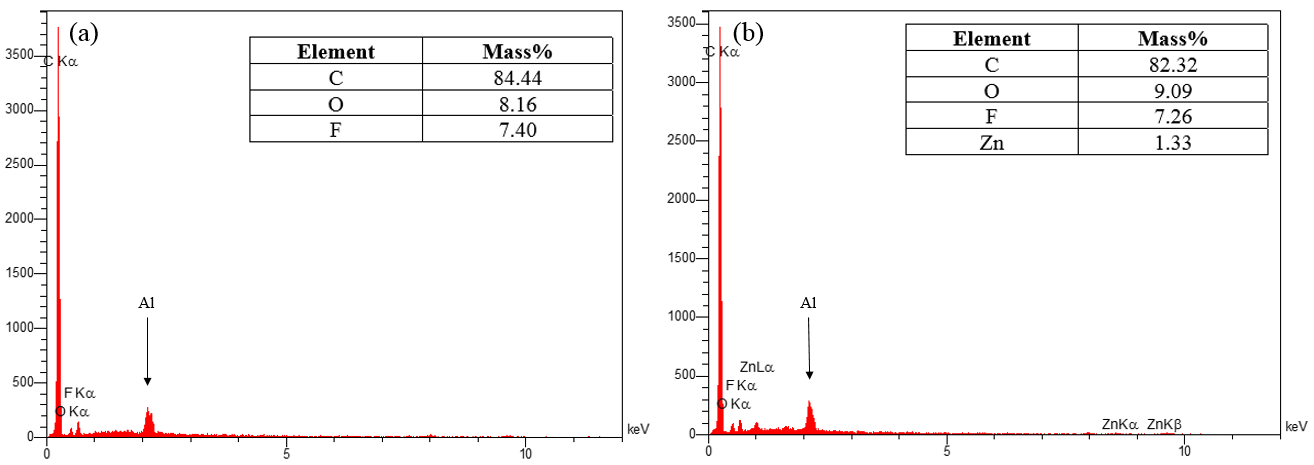


**Figure S9.** EDS test results for (a) E1; and (b) E4 electrodes.


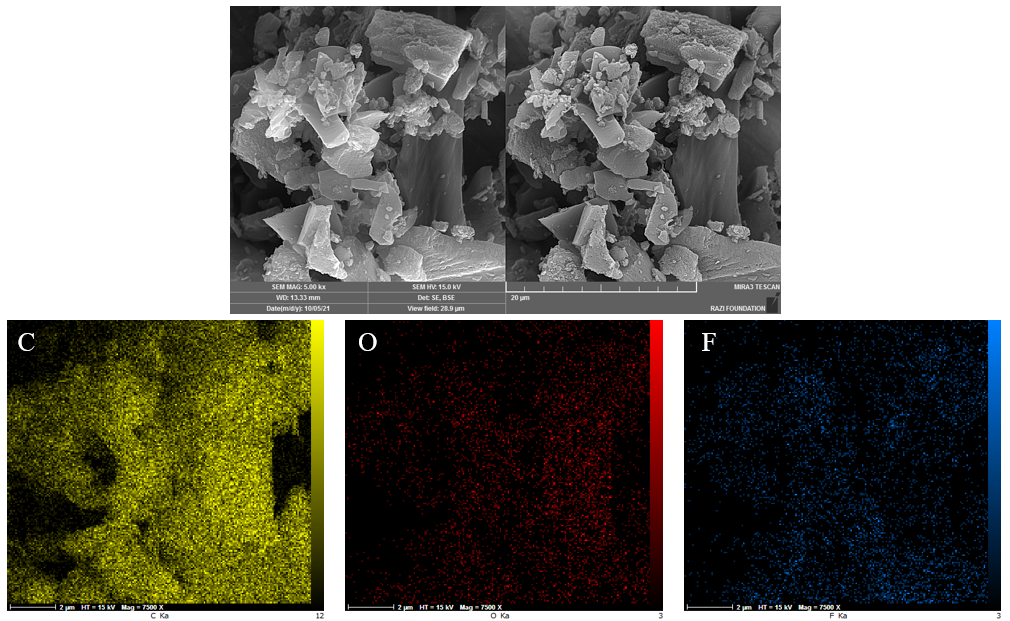


**Figure S10.** Elemental mapping images of E1 electrode.


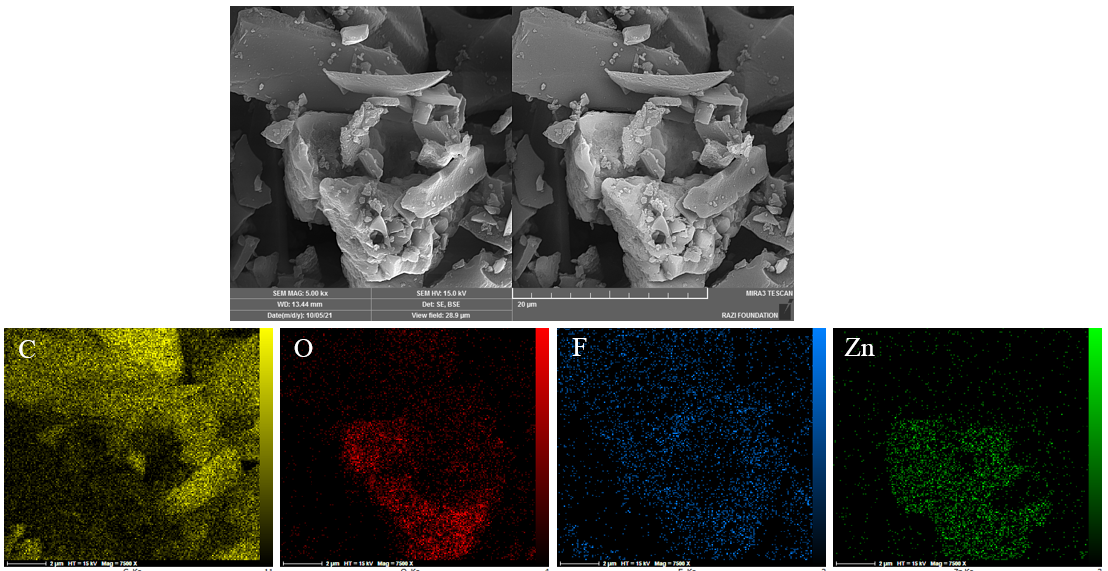


**Figure S11.** Elemental mapping images of E4 electrode.

According to Figure S11, there is a proper distribution of constituent components for the E4 electrode throughout the structure. The utilization of smaller nano-sized Zn-BTC MOF particles promotes better dispersion throughout the electrode structure, while larger micro-sized particles enhance AC particle dispersion in comparison to E2 electrode (Figure S12), ultimately leading to increased ion accessibility to the porous structure of the AC electrode ^9,10^. The presence of fluorine element is due to the presence of PVDF polymer in the electrode structure ^11^. Also, the additional peak observed in both Figure S9 diagrams, which belongs to aluminum, is caused by the aluminum surface of the sample holder ^4,12^.


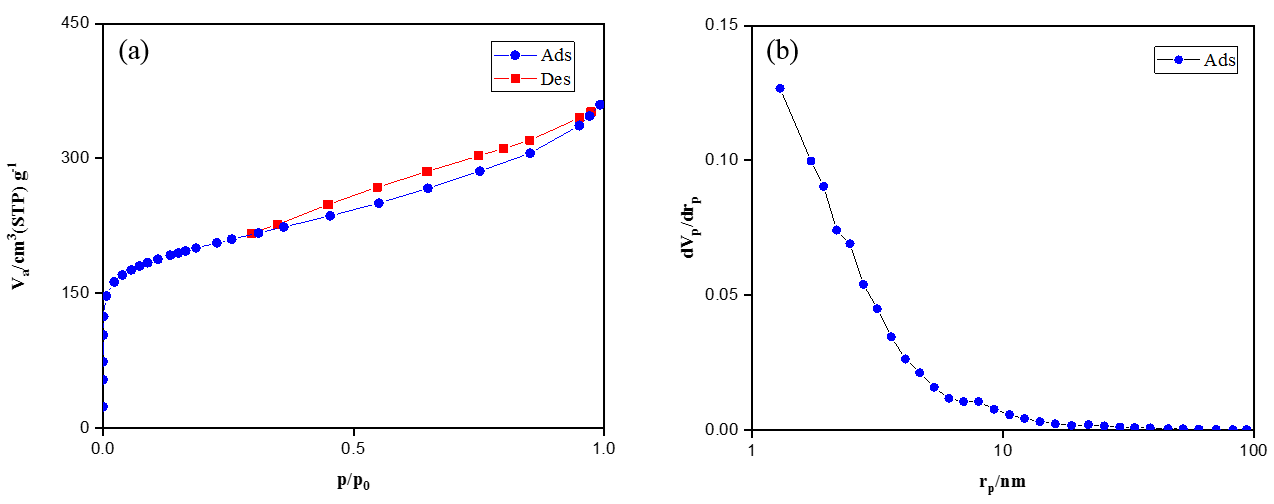


**Figure S12.** (a) Nitrogen adsorption and desorption diagram; and (b) pore size distribution of AC.

**Table S3.** Information from the BET test of AC.

| Material | BET SSA (m^2^ g^-1^) | BJH pores volume (cm^3^ g^-1^) | Mean pore diameter (nm) |
| --- | --- | --- | --- |
| AC | 723 | 0.364 | 3.10 |

**Figure S13.** (a) Desalination results for the SymE1 arrangement at voltages of 1.2 and 1.6 V. (b) Desalination results for the SymE1 arrangement at different flow rates at a voltage of 1.6 V. (c) Desalination results for all three arrangements at a voltage of 1.6 V and a flow rate of 20 mL min^-1^. (d) Bar diagram of SRC and SRE for all three arrangements at a voltage of 1.6 V and a flow rate of 20 mL min^-1^.


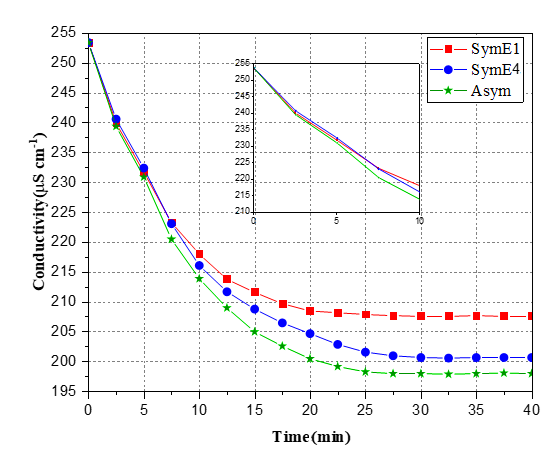

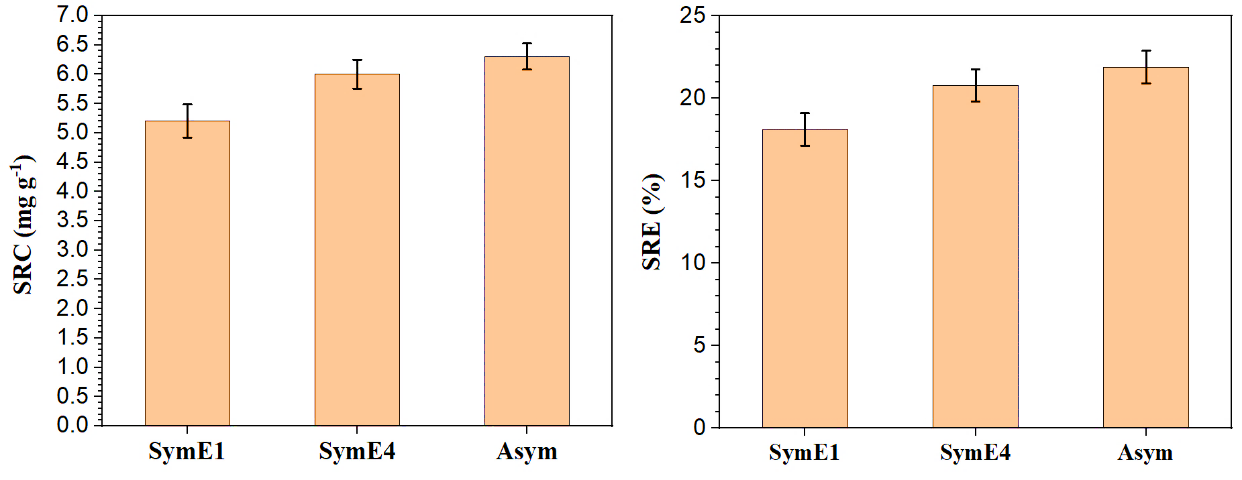


(a)

(b)

(c)

(d)

(e)

**References**

1. Osman, S., Senthil, R. A., Pan, J. & Li, W. Highly activated porous carbon with 3D microspherical structure and hierarchical pores as greatly enhanced cathode material for high-performance supercapacitors. *J. Power Sources* **391**, 162–169 (2018).

2. Lestari, W. W., Inayah, W. C., Rahmawati, F., Larasati & Purwanto, A. Metal-organic frameworks based on zinc(II) and benzene-1,3,5-tricarboxylate modified graphite: Fabrication and application as an anode material in lithium-ion batteries. *J. Math. Fundam. Sci.* **52**, 81–97 (2020).

3. Lestari, W. W., Arvinawati, M., Martien, R. & Kusumaningsih, T. Green and facile synthesis of MOF and nano MOF containing zinc(II) and benzen 1,3,5-tri carboxylate and its study in ibuprofen slow-release. *Mater. Chem. Phys.* **204**, 141–146 (2018).

4. Moloto, W., Mbule, P., Nxumalo, E. & Ntsendwana, B. Stabilizing effects of zinc(II)-benzene-1,3,5-tricarboxylate metal organic frameworks on the performance of TiO2 photoanodes for use in dye-sensitized solar cells. *J. Photochem. Photobiol. A Chem.* **407**, 113063 (2021).

5. Wang, X. *et al.* A zinc(II) benzenetricarboxylate metal organic framework with unusual adsorption properties, and its application to the preconcentration of pesticides. *Microchim. Acta* **184**, 3681–3687 (2017).

6. Jeremy I. Feldblyum, Ming Liu, David W. Gidley, and A. J. M. Reconciling the Discrepancies between Crystallographic Porosity and Guest Access As Exemplified by Zn-HKUST-1. *J. Am. Chem. Soc.* **133**, 18257–18263 (2011).

7. Eddaoudi, M., Li, H. & Yaghi, O. M. Highly porous and stable metal-organic frameworks: Structure design and sorption properties. *J. Am. Chem. Soc.* **122**, 1391–1397 (2000).

8. Yaghi, O. M., Li, H. & Groy, T. L. Construction of porous solids from hydrogen-bonded metal complexes of 1,3,5-benzenetricarboxylic acid. *J. Am. Chem. Soc.* **118**, 9096–9101 (1996).

9. Zhang, Y., Ren, P., Liu, Y. & Presser, V. Particle size distribution influence on capacitive deionization: Insights for electrode preparation. *Desalination* **525**, 115503 (2022).

10. Xing, Z. *et al.* Particle size optimization of metal–organic frameworks for superior capacitive deionization in oxygenated saline water. *Chem. Commun.* **59**, 4515–4518 (2023).

11. Fateminia, R., Rowshanzamir, S. & Mehri, F. Synergistically enhanced nitrate removal by capacitive deionization with activated carbon/PVDF/polyaniline/ZrO2 composite electrode. *Sep. Purif. Technol.* **274**, 119108 (2021).

12. Li, H., Ma, Y. & Niu, R. Improved capacitive deionization performance by coupling TiO2 nanoparticles with carbon nanotubes. *Sep. Purif. Technol.* **171**, 93–100 (2016).
